# A Comprehensive Literature Review on the Role of Bentonite in White Wine Protein Stabilization

**DOI:** 10.3390/foods14233994

**Published:** 2025-11-21

**Authors:** Marco Lagori, Simone Vincenzi, Matteo Marangon, Luca Cattaneo, Maria Alessandra Paissoni, Susana Río Segade, Simone Giacosa, Antonella Bosso, Luca Rolle

**Affiliations:** 1 Department of Agricultural, Forest, and Food Sciences, University of Torino, 12051 Alba, Italy; marco.lagori@unito.it (M.L.); luca.cattaneo289@edu.unito.it (L.C.); mariaalessandra.paissoni@unito.it (M.A.P.); susana.riosegade@unito.it (S.R.S.); simone.giacosa@unito.it (S.G.); luca.rolle@unito.it (L.R.); 2 Department of Agronomy, Food, Natural Resources, Animals and Environment (DAFNAE), University of Padova, 35020 Legnaro, Italy; simone.vincenzi@unipd.it; 3 Interdepartmental Centre for Research in Viticulture and Enology (CIRVE), University of Padova, 31015 Conegliano, Italy; 4 Interdepartmental Centre for Grapevines and Wine Sciences, University of Torino, 12051 Alba, Italy; 5 Consiglio per la Ricerca in Agricoltura e la Analisi della Economia Agraria—Centro di Ricerca Viticoltura ed Enologia, CREA-VE, 14100 Asti, Italy; antonella.bosso@crea.gov.it

**Keywords:** bentonite, grape must and juice, white wine, protein stabilization, turbidity, heat test, alternative approaches

## Abstract

Protein instability leading to haze formation remains a critical challenge in white wine production. For more than a century, bentonite has been the most widely adopted solution due to its effectiveness and efficiency. However, its use poses several drawbacks, including non-specific adsorption of desirable compounds potentially affecting wine quality, wine losses, environmental impact, and health and safety risks for operators. These limitations have spurred extensive research to understand the mechanisms underlying protein instability and to identify alternative stabilization strategies. This review provides a comprehensive analysis of bentonite’s role in white wine protein stabilization, examining its physicochemical properties, treatment variables, and interactions with wine components. Particular attention is given to predictive tests aimed at optimizing dosage, as well as to bentonite’s impact on volatile organic compounds, phenolics, and elemental composition. Furthermore, emerging alternatives and knowledge gaps are discussed to outline future directions toward sustainable and efficient stabilization practices. This synthesis aims to support both scientific advancement and practical applications for the wine industry.

## 1. Introduction

Protein haze formation remains a major concern for wineries [1] since limpidity is an essential sensory quality parameter in white wines [2]. The resulting cloudiness is considered an unacceptable defect by consumers [3,4,5], rendering the affected wines unmarketable [6,7,8], despite taste and smell being unaffected [9].

Bentonite has long been the most effective solution to address this issue [2,8]. Its widespread use is justified by low cost, availability, ease of application, and high efficacy in protein removal [10,11,12]. However, several concerns surround bentonite use in the wine industry [13]. Bentonite sludge causes 3–10% wine loss [14], costing the sector approximately USD 1 billion annually [15]. Despite its affordability, significant costs arise for labor, operating conditions, and waste disposal [1,12,16]. Additionally, bentonite raises both environmental [5,8,17] and safety concerns, particularly related to occupational exposure during handling and disposal, which may pose risks to worker health primarily due to the inhalation of respirable dust containing crystalline silica (a recognized human carcinogen associated with silicosis, pulmonary diseases, and other systemic effects) [13,18]. Furthermore, bentonite also impacts wine quality [19]. Its lack of specificity [20] depletes key sensory compounds, mainly volatile organic compounds (VOCs) [11,20,21,22] and phenolics [23,24,25,26]. It also modifies wine’s elemental composition, releasing or removing elements [27,28] that can influence organoleptic attributes [29].

Concerns over bentonite use [30] have driven decades of research aimed at understanding protein instability, optimizing bentonite application, and developing alternative stabilization strategies [2]. Efficient removal of haze-forming proteins, while minimizing economic, environmental, and quality impacts, remains essential to meet the growing demand for sustainable winemaking practices from both producers and consumers [31,32]. This review consolidates the current knowledge on bentonite’s role in protein stabilization, its impact on wine quality, ways to improve handling through a deeper understanding of protein-haze formation and wine susceptibility, and the latest findings on alternatives approaches, providing insights for both researchers and the wine industry.

## 2. Inside Bentonite: The Structural and Physicochemical Properties Shaping Its Enological Role

Bentonite is a clay-based mineral (hydrous aluminum silicates) primarily composed of montmorillonite, but it also contains accessory minerals such as quartz, chalcedony, feldspars, calcite, dolomite, analcime, pyrite, illite, kaolinite, and gypsum [27,33,34,35]. Its name originates from a rock formation near Fort Benton (MT, USA), although it is globally mined [36].

**Figure 1:**
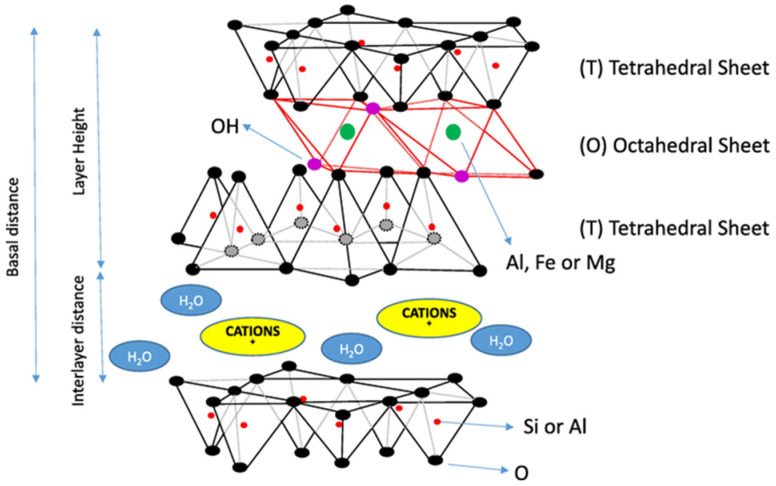
Schematic representation of montmorillonite clay (MMT). Reprinted (adapted) from [37]. Copyright 2021 Gustavo Morari do Nascimento under the terms of the Creative Commons Attribution 3.0 License.

Montmorillonite is a 2:1 dioctahedral smectite that comprises two tetrahedral silicon oxide sheets (Si-O) and one octahedral aluminum hydroxide sheet (Al-O-OH) [34], forming a crystalline layer [27] (Figure 1). The octahedral sites are occupied by Al^3+^. Its isomorphic substitution with divalent cations (Mg^2+^, Fe^2+^) and the intrinsic acidic nature of silica (SiO_2_) lead to a negative charge at wine pH [20,34,35]. This negative charge is largely constant, unaffected by wine composition, although variable charges near hydroxyl groups depend on pH [27].

The negative charge confers adsorption properties [35,38], making bentonite widely used as processing aids in wine production [39], particularly for fining and protein stabilization [13]. Proteins that are positively charged at wine pH [40,41] are adsorbed onto the negatively charged clay surface [5,20,35,42], resulting in adsorption and subsequent removal [5,29,35]. Adsorption also involves van der Waals forces, hydrogen bonds, coordination, and chemisorption [35,43]. This adsorption can occur through mechanisms such as intercalation, delamination, and exfoliation, which may act independently or together [43,44].

The negative charge is counterbalanced by the presence of ions such as Ca^2+^, Na^+^, and Mg^2+^ [45], along with trace amounts of K^+^, Fe^2+^, and Cu^+^, located within interlayer spaces and on particle surfaces [20,46], which also enable ion exchange with the surrounding medium [47]. Notably, the type and ratio of both major and minor cations in bentonite are strongly influenced by its type and geographical origin [27,34,36]. While bentonites may share similar elemental compositions, trace elements vary depending on their source [18]. The relative abundance of the exchangeable cations determines the classification of commercial enological bentonites: natural calcium bentonites, natural sodium bentonites, and sodium-activated bentonites (calcium bentonites treated with sodium carbonate at 80 °C to report similar properties to sodium bentonites) [26,27,36].

**Figure 2:**
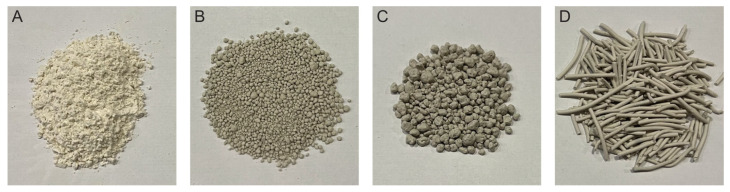
The available product forms of commercial bentonite: (**A**) powder, (**B**) spherical granules (smaller sizes), (**C**) spherical granules (bigger sizes), and (**D**) cylindrical granules.

Morphologically, bentonite has a lamellar structure interspersed with the exchangeable cations and hydration water [42]. Its structural conformation and mineralogical composition confer key physicochemical properties, such as cation exchange capacity (CEC), swelling capacity, and surface charge density [18,28,48]. Swelling behavior, which influences specific surface area (SSA) and adsorption potential, is particularly relevant in winemaking [34,36]. Generally, higher CEC corresponds to greater swelling capacity [48] and thus higher adsorption potential [35]. According to the OIV [33], the montmorillonite purity level should not be below 80% of dry bentonite weight. However, in the context of wine treatment, purity alone is not sufficient to guarantee its effectiveness. Catarino et al. [27] observed that a higher montmorillonite content sometimes correlated with lower SSA and CEC, in agreement with Lambri et al. [20]. The structure and physicochemical properties should also be considered when evaluating the appropriate type of bentonite to use. Additionally, the commercial form of bentonite (powder vs. spherical or cylindrical granules; Figure 2) affects swelling capacity, with powder forms offering higher swelling and adsorbent capacity [33,36]. While higher swelling and SSA enhance protein adsorption, they may also increase the depletion of desirable compounds [20,42,47] and the release of some elements [29], although it seems to be more related to CEC [27]. Element release poses potential quality and safety concerns [49,50], although not all elements of bentonite leach into the wine [36]. In this sense, future research is needed to determine the optimal combination among the composition, physicochemical properties, and product form of bentonites to optimize protein adsorption while reducing undesirable effects. Improved understanding could enhance wine stability, promote more selective formulations, and support sustainable alternatives for the wine industry.

## 3. The Rationale Behind Protein Stabilization in White Wines

### 3.1. The Role of Grape Proteins in Wine Protein Haze Formation

The esthetic wine defect of protein haze [51] occurs when particles at around 1 μm in diameter scatter or block light [34], creating visible cloudiness [39]. This reduces the perceived quality [9,52] and commercial value of wines [47,53].

**Figure 3:**
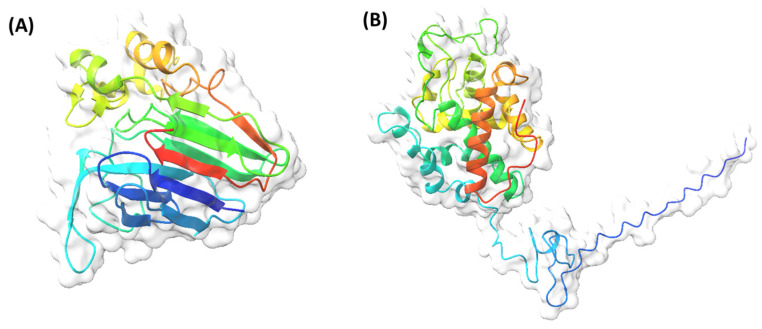
Ribbon diagram showing the overall three-dimensional structures of *Vitis vinifera* unstable proteins: (**A**) thaumatin-like protein (PDB ID:4JRU) and (**B**) class IV chitinase (UniProt ID: Q7XAU6).

The haze-causing compounds are grape proteins known as pathogenesis-related proteins (PRPs) [39], specifically chitinases (CHIs) and thaumatin-like proteins (TLPs) [13,52,54], ranging from 10 to 500 mg/L [55] (Figure 3). PRPs, synthesized after veraison, are found in grape skins and pulps, being involved in defense mechanisms against fungal infections [56,57,58,59,60]. The proteins responsible for wine haze have low molecular masses (<35 kDa) [61], low isoelectric points (IEPs) (4–5) [62] that make them acidic [17], and abundant alpha-helical bonds, classifying them as “soft” proteins [63]. Their compact, globular conformation, stabilized by disulfide bonds, enhances their resistance to proteolysis and acidic pH, enabling them to persist through winemaking and cause haze during wine storage [41,52,57].

While some studies identify CHIs as the primary contributors to instability [54,57,63,64], others also include TLPs [3,9,52,65]. Minor roles are attributed to β-glucanase and ripening-related proteins [3]. The role of TLPs in wine protein haze formation is debated [41]. Initially, both CHIs and TLPs were reported to be implicated in haze [13,39]. Advances in protein purification and analysis have elucidated the specific behaviors of PRPs in terms of melting temperature, unfolding characteristics (reversible or irreversible), and aggregation behavior (representing the factors triggering the instability that will be described in more detail in Section 3.2) [40,66,67]. CHIs are more prone to haze formation due to their lower melting temperature (55 °C) compared to TLPs (56–62 °C) [57], irreversible unfolding behavior, and higher aggregation rate [12,54]. TLPs do not always form aggregates after unfolding, probably because the rate of reversibility of the unfolding is higher than the aggregation kinetics [5,12,54,57,62,66,68]. According to Falconer et al. [57], CHIs’ half-life decreases with temperature: 30 s at 55 °C, 17 min at 45 °C, 1.3 h at 40 °C, 14 h at 35 °C, 4.7 days at 30 °C, and 9 years at 15 °C; by contrast, TLPs exhibit higher stability with a predicted half-life of 45 years at 35 °C [54] in model solutions. The observation that total wine protein concentration usually does not correlate with protein instability [4,9,39,51,54,63,69] confirms differential behaviors among proteins [12,54,66], in addition to matrix effects [9,63,65]. Indeed, a correlation between protein content and the turbidity of heat-induced haze formation (50 °C for 3 h) was found, but only considering CHI content [54]. This supports the hypothesis that CHIs are the main contributors to instability, with increasing CHI content exacerbating the issue [4,5,51,57,63].

Discrepancies in the literature on the role of specific PRPs in protein haze may stem from varying experimental plans (e.g., different heat tests) [12,17], and can be intensified by the different matrix compositions (4), e.g., pH and ionic strengths [12,64], phenolics and polysaccharides [66,68], or sulfate concentration [12,70]. Proteins might be the least variable factor among wines, whereas the matrix varies the most [54]. A further explanation, according to Marangon et al. [54], could be that CHIs, being more unstable, may trigger TLP precipitation under certain conditions, which would otherwise remain soluble [12,57]. Additionally, TLP isoforms may contribute to haze in a variable way [17,41,54,57,62,68]: some TLP isoforms (e.g., 4JRU) behave more like CHIs, having lower melting temperatures and exhibiting less refolding tendency, making them more prone to form visible aggregates [41]. It is also well known that haze-forming proteins vary in composition and content according to the cultivar, grape ripeness, terroir [2,71], vintage [72], disease pressure [73], and harvest conditions [74], as well as the winemaking process and aging, which can lead to the formation of micro-heterogeneities, among other structurally similar proteins (e.g., proteolysis) [5,59], opening up countless possible conditions. In any case, protein type remains the dominant factor in instability dynamics [68]. All of these variables complicate reproducibility and contribute to conflicting results in the literature [34].

Notably, under the current climate change scenario, the content of PRPs in grapes may increase as a consequence of the enhanced stresses that plants experience, thereby exacerbating protein instability issues [75,76]. Indeed, climatic conditions play a fundamental role in determining the protein composition of grapes and, consequently, the future protein stability of wines. Warm and dry conditions during ripening appear to favor instability; however, it is still unclear whether this is due to an increased concentration of the proteins responsible for haze formation or to changes in the overall wine composition [76]. Nevertheless, it has been reported that higher levels of such proteins, and, accordingly, a greater bentonite requirement for stabilization are required in wines obtained from vines that have undergone water stress [75].

### 3.2. Decoding Protein Haze: Dynamics and Wine Instability Predictive Tests

The mechanisms of haze formation involve a complex interplay between protein behavior and non-proteinaceous factors [9,17]. These dynamics are difficult to isolate and study, with protein haze being not yet fully understood [3,51,54,66]. Haze formation is a two-step mechanism: First, a more-or-less reversible loss of conformation (unfolding), which disrupts the tertiary structure [57], forms aggregation-prone intermediates, followed by irreversible aggregation [5,17,51]. Protein unfolding and aggregate formation follow different dynamics: protein unfolding is linked to changes in protein conformational structure, exposing the amino acid side chains normally hidden in the core of the protein, and the hydrophobic sites [41,51,62,66], reducing protein solubility and promoting aggregation [62,66], while colloidal and intermolecular interactions (Lifshitz–van der Waals forces, polar and electrostatic interactions) drive aggregates formation [64], depending on particle size and distance [17]. Noteworthy, the aggregation rate is faster, but the unfolding represents the rate-limiting step for the process [57]. These dynamics are influenced by protein type and content [54,57], storage temperature [17,77], and by the following wine-matrix properties: pH and ionic strength overall [12,62,64], but also polysaccharides, polyphenols, sulfates, metals, and reducing agents [5,9,51,64,65,66,68,70]. Depending on the size and structure, protein aggregation may result in visually undetectable particles (<1 µm), haze, or sediment [5,12,17,54,57,62].

To minimize the drawbacks of bentonite [78], reducing the bentonite dose to essential levels is recommended [22,51]. Predicting instability by determining the optimal bentonite dose for wine stabilization is crucial for the wine industry. For this purpose, stability tests parallel to fining trials are usually used [13]. Protein haze in the stability tests can be triggered by adding reagents, heating, or a combination of both [77], with the aim of accelerating the natural unfolding and precipitation that occur during storage [57,77]. Among predictive tests, the heat test is the most common [64,65] because it appears less drastic [77]. It involves heating, cooling, and turbidity measurement. Turbidity can be assessed visually, spectrophotometrically (absorbance at 520 nm), and nephelometrically, with the latter being the most reliable [13,77]. Various heat test protocols from the literature differ in temperature and heating/cooling times [17]. However, the heat test does not accurately represent the changes and aggregation occurring under actual wine storage conditions, as storage temperatures rarely reach the high levels used in testing [4,63,64]. The heat test, commonly performed at 80 °C lasting from 30 min to 6 h depending on the protocol adopted [2], likely overestimates the required bentonite dose, as most of the proteins, including those stable during storage, are denatured at this temperature [5,51,54,63]. Indeed, attempts to correlate protein content with instability have consistently failed [39,51,54,63,65], likely because some proteins resist thermal induction, while turbidity formation depends on additional factors, as previously indicated [4,51]. Overestimation may also stem from unstable proteins being preferentially adsorbed by bentonite; in fact, a correlation has been observed between the susceptibility of proteins to heat-induced precipitation and their removal by bentonite [5,63]. The proteins are adsorbed by bentonite in the following order: CHIs and β-glucanase, lipid transfer protein (LTP), TLPs, and invertases. The varying tendency for preferential adsorption by bentonite could be attributed to differences in protein IEPs (5–7 for CHIs and β-glucanase; 4–5 for TLPs), with higher values corresponding to proteins carrying a greater positive charge and, consequently, exhibiting stronger electrostatic attraction. However, this phenomenon appears to be more closely related to their intrinsic conformational stability, specifically depending on the α-helix/β-sheet ratio. This protein property affects their ability to undergo conformational changes upon adsorption and lowers the energy required for unfolding [63]. Therefore, the dose should be carefully optimized based on this varying protein affinity. In this context, a predictive test designed to estimate potential haze formation, targeting the proteins most prone to aggregation, could contribute to minimizing the bentonite doses required for effective wine stabilization.

### 3.3. Limitations of Heat Tests in Predicting Protein Instability and Bentonite Dosage

A crucial tool for predicting the bentonite dosage is the heat test, which establishes a threshold to determine wine protein stability [63]. Pocock and Waters [77] found that turbidity becomes visible when the difference in nephelometric turbidity units (ΔNTU) is above 3. Thus, the maximum recommended threshold for a stable wine is 2 ΔNTU [65]. A more accurate estimation of protein instability can be achieved by better understanding the dynamics of protein instability during storage and turbidity development during the heat test [5,64], and therefore correlating heat test results with storage conditions [63,65]. This research gap remains to be addressed. Dufrechou et al. [64] studied the dynamics of protein haze, focusing on pH, ionic strength, and temperature. Unexpectedly, they found that aggregate formation at 25 °C increased as pH decreased, contrary to common findings [9,79], and also increased with ionic strength. Their findings challenge the assumption that increased electrostatic repulsion at lower pH enhances stability, as seen in model solutions [51]. At a lower pH, the electrostatic repulsion destabilizes the protein folded conformation. The effect of ionic strength is more complex; while unfolding is driven by electrostatic repulsion, ionic strength should buffer this effect. After denaturation, increased ionic strength accelerates aggregation by reducing electrostatic repulsion [12,62]. These observations, confirmed in wine, differ in that aggregation in model solutions did not always result in visible cloudiness, while in wine it did. This highlights the influence of other compounds on haze development [3,51,68]. Other studies have suggested that wines with higher pH are more prone to protein instability [9,65,79]. This discrepancy can be explained by the two-step mechanism in protein haze development. Dufrechou et al. [64] highlighted the key role of pH at 25 °C in determining unfolding and aggregation, which occurred only at a lower pH, while a higher pH prevented it. Increasing pH raises the melting temperature of proteins, enhancing stability [63,64]. However, at the high temperatures used in the heat test, pH’s role in unfolding is less significant, because the high temperatures play a primary role. Once the proteins are denatured, aggregation is promoted by reduced electrostatic repulsion at a higher pH [12,64]. Dufrechou et al. [64] also observed that proteins could return to a native or semi-native state post-unfolding at a lower pH, whereas this did not occur at higher pH due to faster aggregation kinetics.

**Figure 4:**
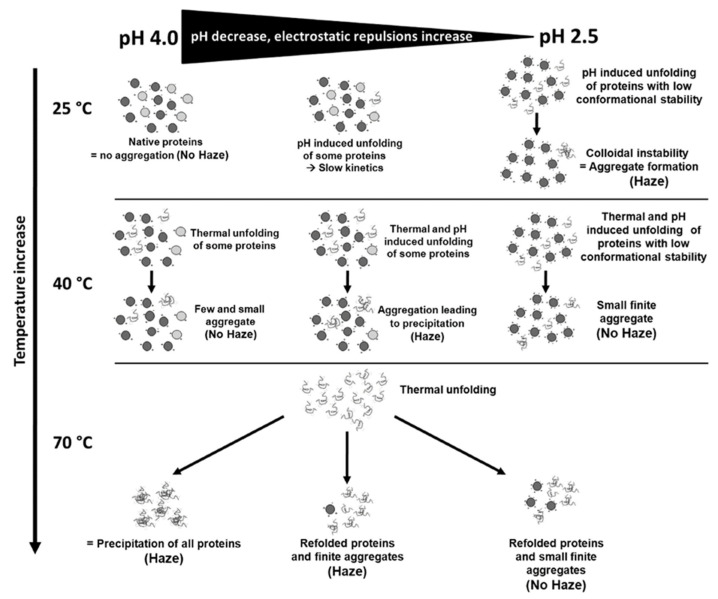
The proposed mechanisms of protein aggregation are influenced by pH and heat treatment, highlighting their combined effect. At ambient temperature, proteins with low conformational stability (e.g., chitinases, β-glucanases, and 22–24 kDa TLP isoforms) precipitate at low pH (≤3.2) due to pH-induced unfolding, driven by increased intramolecular electrostatic repulsions. At 40 °C, both pH and temperature accelerate protein unfolding, leading to aggregation at pH ≤ 3.5, with final haze levels determined by electrostatic repulsions between aggregates (maximized at low pH). At 70 °C, most proteins undergo heat-induced unfolding, and aggregation is primarily governed by intermolecular electrostatic interactions, which diminish as pH increases. Reprinted (adapted) with permission from Dufrechou et al. [64]. Copyright 2012 American Chemical Society.

Figure 4 provides an overview of the protein haze mechanisms proposed by Dufrechou et al. [64]. These findings are consistent with Dordoni et al. [47], who observed that an unstable wine subjected to heat testing showed increased instability with rising temperature and pH (turbidity doubled at pH 3.60 compared to pH 3.00). Vernhet et al. [63] further confirmed the role of pH, comparing two heat tests (40 and 80 °C) on wines with pH values ranging from 2.6 to 4.2. In the 40 °C test, slight variations in ΔNTU with inconsistent trends were observed as pH changed (e.g., from 15 to 30 or from 5 to 20 ΔNTU). Differences in matrix composition may account for this limited variability; however, all wines at pH 4.2 exhibited the lowest ΔNTU. In contrast, at 80 °C, all wines showed a consistent increase in ΔNTU with higher pH, suggesting that, at this temperature, proteins denaturation is independent on pH while aggregation, favored by higher pH, increased accordingly (ΔNTU range: 10–220). Similar observations were described by Lambri et al. [53], who observed no haziness at 80 °C and pH 3.00 in an Erbaluce wine, but haze was already apparent at 60 °C and pH 3.30. The protein tendency to revert to a native or semi-native state due to unfavorable aggregation at low pH has also been observed by other authors [5,12,62,68]. The dual role of pH in the two-step aggregation mechanism may explain why heat testing does not reflect what happens during actual storage conditions [63], as well as why no correlation is found between protein content and instability [63,65] and between ΔNTU and stabilizing bentonite dose [65]. The latter could also be due to the varying efficacy of bentonite shown at different pH levels [63]. According to Dufrechou et al. [64], lower pH may increase protein unfolding or conformational changes, exposing more surface area and hydrophobic sites [41,62,66,80], enhancing interactions with bentonite. An additional explanation could be that the turbidity development depends on the size and shape of the aggregates, which, in turn, are influenced by the characteristics of the matrix, as previously mentioned, and may therefore be wine-dependent. Furthermore, Pocock and Waters [77] suggested that a low level of proteins in wine may not lead to protein haze during short- to medium-term storage, but could still produce haze in predictive tests. Building on these assumptions, Vernhet et al. [63] aimed to correlate heat test results with real storage conditions by simulating accelerated aging (35 °C for one month). They found a higher correlation for the 40 °C (30 min and 4 h) heat tests (R^2^ = 0.64 and 0.67) than for the 80 °C (30 min) heat test (R^2^ = 0.05). A similar result was reported by Pocock and Waters [77]. At 40 °C, only the more unstable proteins (CHIs, some TLPs and β-glucanases) unfold, while stable proteins (TLPs and invertases) do not. This protein behavior aligns with the predicted half-life from Falconer et al. [57]. McRae et al. [65] conducted a similar study to correlate the bentonite dose predicted by two heat tests (a more drastic one with 6 h at 80 °C and 18 h at 4 °C, and a less drastic one with 2 h at 80 °C and 3 h at 20 °C) with storage trials of wines at 17 and 28 °C for one year. The doses predicted by the more drastic test led to stable wines at both storage temperatures, while those predicted by the less drastic test resulted in slightly hazier but still stable wines at 17 °C. On the contrary, at 28 °C, not all wines were stable, highlighting the importance of considering the matrix effect in addition to the test results, as it may influence bentonite treatment’s effectiveness and haziness during storage.

Dufrechou et al. [62] reinforced the theory that heat testing does not replicate actual wine storage conditions [17,63,64], noting that pH-induced conformational changes in CHIs affect the protein structure locally without altering ellipticity, unlike temperature-induced changes, which unwind α-helices, leading to protein unfolding and reduced ellipticity; thus, pH appears to only affect local changes in CHIs without altering their secondary structure [62].

Given the above, the heat test must be precisely defined and the impact of various variables thoroughly examined. Heat test conditions can significantly influence the results, potentially leading to the over- or under-dosing of bentonite [65]. Proteins’ conformational stability in a given solvent is rather weak and is strongly influenced by even minor variations in external conditions [5]; thus, small changes in assay conditions (e.g., temperature and time) can also impact the results [65,80]. Outcomes are highly dependent on the specific conditions of the test [3,63,65]. As Pocock and Waters [77] and McRae et al. [65] suggest, attention should also be given to test methodology and procedure standardization (e.g., temperature, heating/cooling times, sample volume, method, and stability threshold) to ensure consistency and reproducibility. Given the inherent imperfections of the heat test, the ideal approach to overcome these limitations would be to correlate the content of haze-forming proteins with the potential protein instability of a given wine. By creating a database containing this information for a large set of wines, alongside the key compositional parameters of said wines (e.g., pH, ethanol content, acidity), would enable the development of regression models. By inputting parameters such as CHI and TLP concentrations, pH, and alcohol content, these models could predict the risk of protein instability and determine the necessary bentonite dosage.

Another possible approach, if the predominant role of specific proteins (e.g., CHIs, β-glucanases, and certain TLP isoforms) in instability is confirmed, could be the development of targeted products for these specific proteins to overcome the limitations of predicting bentonite dosage.

## 4. Bentonite in Winemaking: The Key Factors Influencing Its Effectiveness

In winemaking, bentonite functions as both a fining agent to clarify wines or a stabilizing agent against protein haze. Its versatility allows for its use at various stages, from initial must clarification to the final stabilization prior to bottling [13].

Bentonite, in its various commercially available forms (Figure 2), is pre-hydrated in water prior to use. This key step allows for the clay structure to swell, thereby increasing the surface area available for interactions and determining the efficiency of the subsequent treatment. Bentonite addition involves dispersion, adsorption of solutes and colloids, and sedimentation of the resulting complexes [81]. While protein adsorption is rapid, cellar practice usually requires 1–2 weeks for stable precipitate formation and racking [13]. The duration depends on tank volume and sedimentation. Faster techniques such as filtration or centrifugation can shorten the process, increase wine recovery, and improve sustainability [82], although possible oxidative effects may reduce quality [83].

As a non-specific adsorbent, bentonite can also remove desirable compounds (e.g., VOCs and phenolics). Minimizing these losses requires knowledge of both bentonite properties (intrinsic factors) and wine matrix characteristics (extrinsic factors). A comprehensive understanding of these parameters is essential to optimize bentonite application, minimize quality losses, and ensure sustainability [47].

### 4.1. Intrinsic Factors

The physicochemical properties of the clay largely determine its effectiveness in protein stabilization and its impact on wine quality [6,16,18,20,34,42,47]. Catarino et al. [27] and Lambri et al. [20] reported that bentonites with a higher Na^+^/Ca^2+^ ratio are more efficient, showing an improved relationship between the applied dose and the amount of protein removed. This is because they may hydrate more [36], increasing surface area and electrostatic interactions [35]. However, treatments performed by Pargoletti et al. [34] showed that the bentonite with the highest Na^+^/Ca^2+^ ratio did not result in the highest protein removal. Similarly, Salazar et al. [16] stated that activated sodium bentonite was more effective than natural sodium bentonite. The role of SSA, CEC, and montmorillonite content may be more significant than the Na^+^/Ca^2+^ ratio [10]. For instance, Lambri et al. [20] found that the bentonite with the highest Na^+^/Ca^2+^ ratio had four times lower SSA, reducing adsorption power. SSA is often considered the most critical parameter [10,20], although some authors argue that CEC is more influential [27,35]. Treatment efficiency likely results from a synergy of these factors [48].

### 4.2. Extrinsic Factors

The variability in results across studies suggests that intrinsic factors alone cannot fully explain bentonite treatment outcomes. In addition to the physicochemical properties of bentonite, extrinsic factors must be taken into account, since they significantly influence the treatment. As shown in protein studies, factors like pH [62,64], ionic strength [12,70], tannins [3,66], polysaccharides [20,68], and protein content and composition [5] can influence macromolecule behavior, reinforcing that the matrix can also affects protein stabilization by modulating protein affinity for bentonite [42]. The medium characteristics can influence bentonite’s behavior by modulating its structural conformation [34]. In the transition from a water–clay suspension to wine, the change in pH alters the ionic strength. This variation in ionic strength leads to the compression of the bentonite double layer and reductions in the dominance of edge-to-face attractions, resulting in the breakdown of the house-of-cards-like structure [84]. Dordoni et al. [47] observed a greater protein removal efficiency in clays exhibiting a less pronounced collapse of the double layer, suggesting distinct physicochemical behaviors among individual clay types, for which structural stability appears to be a key factor.

According to Xifang et al. [35], wine pH influences the treatment the most by affecting bentonite’s surface charge and protein ionization. However, the literature on pH’s effects shows significant discrepancies. Some studies suggest higher treatment efficiency at a lower pH [64,85] due to stronger electrostatic attraction between positively charged proteins and negatively charged bentonite particles [35], while others report higher protein removal at a higher pH [47]. For instance, Lambri et al. [20] found greater protein removal at pH 3.60 than at 3.30. Lambri et al. [10] observed higher adsorption in cv. Chardonnay wine at pH 3.42 compared to cv. Sauvignon wine at pH 3.26, and Catarino et al. [27] noted higher adsorption at pH 3.80. They attributed this behavior to less competition from H^+^ ions, for which bentonite has a high affinity, and a favored K^+^ exchange at higher pH [28,47]. As K^+^ has a mean volume diameter more than twice that of H^+^ [85], it increases interlayer space and thus the available surface area for interactions.

The role of ethanol in protein adsorption remains uncertain. Generally, moving from an aqueous to a hydroalcoholic solution increases protein adsorption [34,38]. Ethanol does not affect the interaction at alcohol contents between 10% and 13% [85], but Lambri et al. [20] observed greater bentonite efficiency at higher pH values moving within a broader alcohol range (11–14.2%). An interesting result was reported by Xifang et al. [35], who observed that protein absorption in a model wine increased as the alcohol concentration rose from 4% to 12% by volume, then decreased dramatically as the concentration increased to 20%.

Polysaccharides and mannoproteins have been shown to reduce treatment efficiency by acting as protective colloids, preventing protein interactions with the clay [20].

Protein content [20] and type [42] also impact bentonite efficiency, which decreases with higher protein concentrations [8]. This aligns with studies indicating that certain proteins have higher affinity for bentonite, while others remain in solution even at higher doses [5,10,47,63]. Jaeckels et al. [86] found that glycoproteins were minimally affected by bentonite, while 96% of chitinases (CHIs) were adsorbed. The greatest variability was observed among thaumatin-like proteins (TLPs) isoforms, which showed removal rates ranging from 0% to 98% [86], likely due to differences in protein hydrophobicity [41]. These findings regarding the specific affinity between different proteins and bentonite are relevant for the quality characteristics of wine [38]. Selectively removing proteins involved in haze formation, while preserving those that are not, would be valuable for improving wine quality. For example, proteins, together with polysaccharides, have been shown to enhance foamability in sparkling wines [87,88]. Additionally, proteins may play a role in increasing the intensity of aromas, especially when aroma compounds are present at low concentrations, although the underlying mechanism remains unclear. It has been suggested that protein binding with aroma compounds may reduce the suppressive effect that odorants tend to exert on each other in mixtures [89].

Temperature also influences bentonite’s protein adsorption capacity, with higher temperatures (25 °C) enhancing adsorption compared to treatments conducted at lower temperatures (5 °C) [85], in agreement with Xifang et al. [35].

Another crucial aspect is the timing of bentonite addition [8]. Compared to earlier studies, more recent ones suggest that adding it during alcoholic fermentation (regardless of whether this occurs at an early, mid, or late stage) is more effective than adding it either before or after fermentation [8,16,42,90]. Pocock et al. [8] found that pre-fermentation treatment is the least effective, requiring higher amounts of bentonite. The bentonite’s efficiency is likely reduced because, in grape juice, it also binds to proteins that are not involved in haze formation—proteins that are either stable or would precipitate naturally during fermentation [52]. In addition, a higher alcohol content in later fermentation stages increases bentonite effectiveness by expanding the interlayer space. Greater efficiency during fermentation could also be attributed to the higher K^+^ concentration in this winemaking phase compared to later stages, which induces swelling [85], enhancing the surface area and protein adsorption. The reduction in bentonite doses required when added during fermentation stages varies from 14 to 16% and from 19 to 21%, depending on the stability test applied [42,90], up to 50% in some cases [11], in agreement with other studies [41,51,85]. This leads to fewer negative impacts on quality, economics, and the environment [8]. The lees become more compact, increasing wine recovery [8], and fewer interventions (e.g., rackings) are needed, which could otherwise reduce wine quality and delay its market release [90].

This interplay between bentonite characteristics, medium, and timing explains the complexity in determining the exact impact of each factor on protein adsorption [34]. Given the variability and the well-documented fact that different bentonites, and even different brands of the same type, yield different results, the current best advice is to conduct stabilization tests with various products to identify the one most suitable for the specific needs of the wine being treated [10].

## 5. How Bentonite Influences Volatile Organic Compounds (VOCs)

VOCs, essential for wine quality, are classified into primary, secondary, and tertiary aromas depending on their origin during the wine production process [91]. Their complex interactions shape the wine’s sensory profile [92], yet the impact of bentonite remains insufficiently understood. As a non-selective adsorbent, bentonite may also alter the aromatic bouquet by interacting with VOCs [6,20,21,90]. The interaction between VOCs and bentonite can occur through different mechanisms, although the extent to which this involves the direct or indirect removal of VOCs is not yet fully understood. Table 1 provides a summary of the principal findings reported in the literature regarding the impact of bentonite on VOCs. Voilley et al. [93] first demonstrated the direct adsorption of VOCs in model solutions, while later studies hypothesized additional interaction mechanisms. Vincenzi et al. [22] investigated the relationship between VOCs, proteins, and bentonites by examining their behavior in wine and model solutions. In wine, bentonite treatment led to a general loss of VOCs, particularly ethyl esters and fatty acids. The reduction was more pronounced for ethyl esters with longer hydrocarbon chains, ranging from 20% loss for ethyl butanoate to 84% for ethyl dodecanoate, suggesting that hydrophobicity, along with weak interactions such as van der Waals forces and hydrogen bonding, plays an important role. The addition of exogenous proteins further increased this loss, particularly for ethyl esters and fatty acids, supporting the hypothesis of indirect removal [20,21]. To further elucidate these interactions, Vincenzi et al. [22] tested bentonite treatment in a model solution, adding selected ethyl esters with and without grape proteins (CHIs and TLPs). The results confirm a synergistic effect between bentonite and the PRPs in the removal of VOCs. Interestingly, this effect was more pronounced in CHI-containing model solution, reinforcing the role of hydrophobic forces in this interaction, as CHIs are more hydrophobic than TLPs [40]. Notably, bentonite exhibited a selective affinity for ethyl esters and fatty acids, whereas its impact on terpenes was negligible, even in the presence of proteins [22]. Di Gaspero et al. [94] confirmed that grape proteins interact with VOCs, indirectly facilitating the removal of these compounds after bentonite treatment. They investigated the UV-photo and thermal stability of a specific TLP (VVTL1) in a model wine, with and without four ethyl esters (ethyl hexanoate, ethyl octanoate, ethyl decanoate, and ethyl dodecanoate). The UV-denaturation rates indicated an interaction between VVLT1 and the esters, which enhanced the protein’s photostability. Ethyl octanoate formed the most stable complex, followed by ethyl decanoate, ethyl dodecanoate, and ethyl hexanoate.

The wine style can also influence bentonite effectiveness. Lambri et al. [20] studied bentonite’s effects on two Chardonnay wines with or without a six-month lees aging. The wine aged on lees had a higher protein concentration due to yeast autolysis, which released proteins and peptides. They observed that the difference in protein concentration influenced the efficiency of bentonite in removing proteins. Specifically, the wine with a lower protein concentration exhibited a higher percentage of protein removal. This finding is consistent with Achaerandio et al. [38], who observed that the adsorption isotherm of the protein–bentonite system demonstrated greater adsorption at lower solute concentrations. They concluded that the reduced percentage of protein removal in the wine aged on the lees was attributable not only to its higher protein content, but also to the protective effects of polysaccharides and mannoproteins released by the yeasts. This protective effect, exhibited by yeast-derived macromolecules and colloids, has also been observed in relation to VOCs. Yeast cell wall proteins are glycosylated [95], which confers stability by carrying negative charges within the wine’s pH range. These molecules can interact with VOCs, leading to electrostatic repulsion from bentonite, as both carry the same electrostatic charge. This repulsion prevents the interaction between VOCs, bound with these molecules, and bentonite, ultimately inhibiting their removal. However, they observed specific behaviors among VOCs, proteins, and bentonite, governed by complex interactions. Bentonite–VOC binding can be direct or indirect: some compounds bind to proteins via hydrogen bonds and are later removed by bentonite, while high protein concentrations may prevent this interaction by promoting protein–protein binding [81]. Interestingly, the protective effect was not observed in the study performed by Vincenzi et al. [22], in which sugar-containing molecules extracted from a cv. Manzoni Bianco wine and attributed to yeast-derived mannoproteins were added to the samples. The protective effect of certain macromolecules may be compound-specific. In addition, different bentonites exhibit distinct behaviors, also with dose-dependent effects. Although these dynamics are difficult to define, VOC hydrophobicity remains a key factor, especially for esters and fatty acids.

The timing of bentonite addition is a crucial factor in wine quality. Key VOCs, such as esters associated with fruity and floral aromas, are synthesized during fermentation and they are influenced by yeast metabolism, as well as by the amino acid profile and content. Regarding the latter, changes in composition and nitrogen content can potentially have either a positive or negative impact on final wine quality [96]. Since bentonite can reduce the amino acid content in the grape must [97], it affects the yeast-available nitrogen (YAN) while leaving ammonium ion levels unaltered, likely due to its higher solubility in must and weaker association with the insoluble substances that are removed by bentonite. Consequently, bentonite can indirectly influence wine bouquet by altering the synthesis of VOCs. Burin et al. [97] examined the impact of pre-fermentative clarification on cv. Chardonnay grape must using bentonite, silica, and a pectinolytic enzyme. Bentonite-treated must exhibited the lowest YAN levels. A positive correlation between YAN and the synthesis of total esters was confirmed, in agreement with other authors [98,99]. The wine fined with bentonite before fermentation had the lowest total ester concentration, but interestingly showed the highest concentration of ethyl esters, suggesting a selective effect on different ester families. Additionally, bentonite led to a reduction in varietal compounds such as terpenes and C-13 norisoprenoids. This was attributed to both the direct interaction between bentonite and the glycosylated precursors and the indirect adsorption of these compounds onto precipitated solids, in agreement with Moio et al. [100] and Armada and Falqué [101]. The lower terpene concentration may also stem from YAN reduction, as higher YAN availability enhances monoterpenes synthesis by yeasts [102]. However, given the limited literature on the YAN’s role in isoprenoid metabolism, their reduction is more plausibly linked to bentonite’s affinity for the glycosylated precursors, which are removed during clarification. Lambri et al. [21] investigated the impact of bentonite on terpenols in both must and wine from cv. Moscato bianco. Their findings indicate that bentonite had no significant effect on free terpenols in the grape must compared to the untreated control, supporting the hypothesis that bentonite primarily interacts with glycosidic precursors. However, the most notable aspect of this study was the inconsistency of outcomes across vintages. In the 2006 vintage, the highest terpenols concentration was observed in the untreated control, followed by the wine treated at the grape must level, the wine subjected to double treatment (pre- and post-alcoholic fermentation [AF]), and, finally, the wine treated solely post AF. In contrast, in the 2007 vintage, the highest terpenols concentration was found in the wine treated solely post AF, followed by the double-treated wine (pre- and post AF), the control, and, lastly, the wine treated only pre-AF. This highlights a pronounced vintage effect, underscoring the critical role of matrix composition differences in determining the outcomes of bentonite treatment, as previously discussed in Section 4.2. Sanborn et al. [103] found no significant differences when bentonite was added to cv. Chardonnay wine, but observed some variations in cv. Gewürztraminer wine. However, these differences were not sensory perceived. Horvat et al. [90] also reported a negative impact on certain free monoterpenes, while the effect on bound terpenes during fermentation ranged from mild to moderate, despite their experimental conditions involving particularly high bentonite dosages, ranging from 185 to 260 g/hL, administered through an initial addition of 100 g/hL at various stages (pre-fermentation, onset, during, and at the end of fermentation), followed by a final dose for complete stabilization. The variety, timing of addition (grape must or wine), and dosage of bentonite play a key role. It is plausible that low bentonite doses do not significantly affect varietal grape compounds, especially when added to wine [22] and, in general, the dosage plays a crucial role in determining the extent of VOC losses [20]. When lower dosages are applied, the removal of most VOCS appears negligible.

In addition to the previous considerations, the indirect role of bentonite on VOCs through its influence on enzymatic activity should also be acknowledged, which contributes to further controversy in the published effects. Bentonite exhibits a strong affinity for β-glucosidase [104], significantly greater than for CHIs and TLPs, likely due to β-glucosidase’s greater structural flexibility, which results from the absence of disulfide bonds that stabilize other proteins. β-glucosidases are crucial in the release of monoterpenes by hydrolyzing glycosidic bonds, thus enabling their volatilization [105,106]. Therefore, performing bentonite treatments on the grape must when performing a vinification of varietal VOC-rich grape varieties may not be the optimal choice, as it removes both glycosylated compounds and the enzyme responsible for releasing the aglycone necessary for their perception. Nonetheless, Horvat et al. [90] highlights that the addition of bentonite during mid or late fermentation resulted in wines with higher concentrations of VOCs, particularly fermentative acids and esters, in agreement with Lira et al. [11]. This effect could be attributed to both preservative and stimulatory factors: bentonite may safeguard esters by inhibiting the enzymes that are particularly active during the final stages of fermentation [107], such as esterases, which adversely affects their content while also enhancing the esters’ synthesis, as observed by Lukic et al. [108], who reported that the presence of suspended solids, subsequently precipitated by bentonite, inhibits this process. This multifaceted effect of bentonite, encompassing removal, protection, and synthesis stimulation, is also supported by Lira et al. [14], who observed higher concentrations of fermentative compounds (ethyl esters, acetates, and fatty acids) in cv. Macabeo wines when bentonite was added at the end of fermentation, in agreement with Salazar et al. [16]. More recently, Lukić et al. [6] confirmed that bentonite addition during fermentation enhances VOC content, particularly fatty acids and ethyl esters, compared to untreated control. As previously hypothesized, it is plausible that the reduction in suspended solids, compared to the untreated control, favored their synthesis, or that bentonite limited the action of the enzymes that negatively affect their content.

The type of bentonite also influences the effect on VOCs. Lambri et al. [109] examined the interactions between different bentonite types and VOCs under model conditions, excluding wine macromolecules. They confirmed that adsorption occurs either via electrostatic or physical mechanisms, depending on the bentonite’s properties. A lower SSA and higher charge density were associated with reduced adsorption, whereas a higher SSA, greater Na^+^/Ca^2+^ ratio, and lower charge density enhanced adsorption, particularly for esters. Nonetheless, adsorption dynamics vary within compound classes and depend on molecular characteristics, primarily hydrophobicity, solubility, and size. Salazar et al. [16] investigated the effect of two different types of bentonites (sodium bentonite and sodium-activated bentonite) added at different winemaking stages. When added to the grape must at the same dosage, the sodium-activated bentonite had a lower impact on VOCs than sodium bentonite. However, this trend reversed when bentonite was added during fermentation. Towards the end of the AF, sodium bentonite had a milder effect on VOCs than sodium-activated bentonite. The authors suggested two possible explanations: sodium-activated bentonite either directly removed more VOCs, or its binding sites interfered with yeast metabolism. In contrast, Wimalasiri et al. [110] found no significant differences in the impact of different bentonite types on 36 VOCs on the grape must from red cv. Pinot noir treated with calcium bentonite, sodium bentonite, and sodium–calcium bentonite. Only ethyl cinnamate, hexyl acetate, and cis-3-hexenol were affected. However, as this experiment was performed on red grape must, polyphenols and other skin-derived compounds may have influenced bentonite–VOC interactions. In red wines, all proteins bind to tannins, forming protein-tannin sub-aggregates stabilized in solution by polysaccharides, creating a colloidal system. Consequently, macromolecule–VOC interactions differ significantly from those in white wines. Lukić et al. [6] confirmed that different types of bentonites exhibit varying affinities toward wine molecules, including proteins and VOCs, leading to distinct effects across all of the analyzed compound classes. Notably, linalool concentrations were higher in all bentonite-treated wines compared to the untreated control, whereas hydrocarbon monoterpenes, such as β-pinene and 3-carene, significantly decreased, suggesting a structural influence on adsorption. Other terpenes, such as citronellol, geraniol, α-terpineol, and nerol, exhibited varying interactions depending on the bentonite type. Similarly, fatty acids and esters displayed consistent molecule-specific response to different bentonites. On average, the sodium-activated bentonite combined with adsorbed silica and activated silica had the most favorable impact on VOCs with respect to an activated sodium bentonite and an active sodium–calcium bentonite.

The impact of bentonite on wine VOCs remains debated and not yet fully elucidated. Its effects depend on various factors, including bentonite type and characteristics, timing of addition, dosage, wine matrix, and individual molecule characteristics. However, a deeper investigation into these dynamics could help to mitigate its negative impacts while leveraging its benefits, particularly in relation to the specific grape variety being vinified and its aromatic profile.

**Table 1:** Extraction table of the main findings on the effects of bentonite on volatile organic compounds (VOCs).

Author (Year)	Matrix	Bentonite Type and Dosage	Main Findings
Lukíć et al. [6]	Cv. Malvazija istarska grape must near the end of the fermentation and wine	Granular activated sodium bentonite (GSAB) 95 g/hL, activated sodium bentonite (PEN) 95 g/hL, activated sodium bentonite with specifically adsorbed silica and activated silica (MVC) 143 g/hL, active sodium-calcium bentonite (PUR) 238 g/hL	Bentonite types affected VOCs during fermentation selectively: linalool increased, notably with GSAB and PEN; and β-pinene, 3-carene decreased. GSAB enhanced citronellol and geraniol; PEN increased α-terpineol and nerol. Volatile fatty acids were highest in PUR and PEN. Ethyl and acetate esters showed the highest values in PEN, MVC, and PUR. Subsequent bentonite clarification reduced monoterpenols and β-damascenone and increased specific fatty acids and esters; acetate esters remained high in MVC, PUR, and PEN.
Wimalasiri et al. [110]	Cv. Pinot noir grape must, addition before cold soaking	Sodium, calcium, and sodium-calcium combined bentonite 50 g/hL	Bentonite had a minimal effect on Pinot noir aroma. Only ethyl cinnamate, hexyl acetate, and cis-3-hexenol significantly decreased.
Horvat et al. [90]	Cv. Malvazija istarska grape must before/during fermentation and wine (JU: clear juice; BE: beginning of fermentation; MD: middle of fermentation; EN: near the end of fermentation)	Granular activated sodium bentonite 100 g/hL initial dose at different winemaking phases, with the additional dose to achieve complete stabilization, the total dosage ranged from 185 to 260 g/hL	Bentonite during fermentation reduced citronellol and free geraniol. An additional fining step increased some monoterpenes via hydrolysis and precursor oxidation. β-Damascenone was higher in BE, MD, and EN than JU, but decreased after additional fining. Bentonite inhibited enzymes forming C6 alcohols; 1-octen-4-ol and benzaldehyde increased with additional fining. Bentonite retained more fermentation acids and esters (especially acetates), although esters decreased after final fining. Bound volatile compounds were moderately affected by bentonite.
Salazar et al. [16]	Cv. Sauvignon blanc grape must and wine (before fermentation, early fermentation, late fermentation, and after fermentation)	Sodium bentonite (150 g/hL, sodium-activated bentonite (250 g/hL)	Sodium versus sodium-activated bentonite effects varied with dose and timing: pre-fermentation addition of sodium-activated bentonite yielded similar or higher VOC levels than sodium bentonite. During fermentation, sodium bentonite led to higher VOC concentrations. Late-fermentation fining enhanced protein stability, but increased VOC losses, particularly with sodium-activated bentonite; however, fining during fermentation retained VOC levels comparable to or higher than the controls.
Di Gaspero et al. [94]	Model wine solution	-	UV-photo and thermal denaturation assays showed that ethyl esters bind VVTL1, with chain-length-dependent effects on protein stability, indicating that bentonite fining can indirectly alter wine aroma via protein removal.
Burin et al. [97]	Cv. Chardonnay grape must	Activated bentonite (7 mL/L of bentonite solution 10% m/v), pectinolytic enzyme (1 mL/L), silica sol (2 mL/L)	Bentonite decreased YAN and amino acid content. Enzyme-treated must led to wines with the greatest levels of terpenes, C13-norisoprenoids, and total esters. Bentonite wines showed the lowest total esters but the highest ethyl ester concentrations, while enzyme wines were richest in acetate esters, except phenylethyl acetate. Bentonite wines showed the lowest hydrogen sulfide and methionol contents. Based on Principal Component Analysis (PCA), nitrogen availability and volatile profile were associated with enzyme, increasing varietal aromas, bentonite with enhanced ethyl esters, and silica with reduced VOCs.
Vincenzi et al. [22]	Model wine solution	Activated sodium bentonite 10 g/hL	Bentonite alone minimally affected monoterpenes, but effectively removed ethyl esters and fatty acids, with removal efficiency increasing with chain length. Purified CHIs and TLPs synergistically enhanced long-chain ethyl esters removal via hydrophobic interactions, whereas yeast mannoproteins had no protective effect. Bentonite’s impact on grape-derived VOCs was low, except for β-damascenone. The primary mechanism is direct adsorption, especially for fermentation-derived aromas.
Lira et al. [11]	Cv. Albariño grape must and wine (before, at the beginning, in the middle, and at the end of fermentation)	Sodium granular bentonite 40 g/hL	Bentonite timing during fermentation affected ester and acid contents. Total terpene levels were highest in control and end-fermentation treatment and lowest in clarified musts. Wines fined during fermentation were clearly separated from control and clarified-must samples by PCA. Sensory analysis favored mid- and late-fermentation fining for enhanced aroma intensity and quality.
Lira et al. [14]	Cv. Macabeo wine in pilot and industrial scale (before, at the beginning, in the middle, and at the end of fermentation)	Activated bentonite 25 g/hL	Bentonite addition affected VOCs: At the pilot scale, ethyl ester and acetate contents were the highest when added at the end of fermentation or during must clarification and were the lowest when added during fermentation. At the industrial scale, grape must clarification yielded lower ethyl esters and acetates. The ethyl ester-to-acetate ratio was comparable to controls, except for a 13% increase with end-fermentation addition at the pilot scale and a 25% decrease with must clarification at the industrial scale. Fatty acids were reduced by bentonite at the pilot scale; industrial-scale must clarification and end-fermentation addition increased fatty acids, but addition during fermentation decreased them.
Lambri et al. [109]	Model wine solution	Activated sodium bentonite (A and B same montmorillonite, A powder, B granular. C montmorillonite containing magnesium smectite, powder), 20, 50, and 100 g/hL	SSA, charge density, Na^+^/Ca^2+^ ratio outweigh the VOC characteristics in determining adsorption. Bentonites A and C, with lower SSA and higher charge densities, primarily remove hydrophobic molecules through physical adsorption, with Bentonite C also effective at removing ionic compounds. Bentonite B, with higher SSA and lower charge density, exhibits strongest adsorption of ethyl esters and the highest adsorption capacity and intensity. Most compounds showed slightly unfavorable adsorption (*n* < 1), consistent with physical interactions, while favorable adsorption (*n* > 1) implied chemical bonding.
Lambri et al. [21]	Cv. Moscato bianco grape must and wine from two vintages (2006–2007)	Granular activated sodium bentonite 100–200 g/hL	Bentonite reduced both free and bound terpenes: free linalool, α-terpineol, and citronellol by 16 to 30%; and glycosylated linalool, nerol, and geraniol by up to 49%. During fermentation, free terpenols rose most in untreated 2006 wines. In bottled 2006 samples, free terpenols levels ranked as control > must-treated > double-treated > post-fermentation treatment; in 2007, post-fermentation treatment yielded the highest concentrations. Vintage exerted a greater influence than bentonite treatment.
Lambri et al. [20]	Cv. Chardonnay wine	Three different activated sodium bentonite, 20, 50, and 100 g/hL. Wine A not aged on lees, wine B aged 6 months on lees	Bentonite significantly affected 26 aroma compounds mainly via protein removal rather than direct adsorption. In wine A, rich in grape-derived proteins, ethyl butyrate and ethyl hexanoate were more readily removed, while yeast-derived proteins in wine B had a protective effect. Compound hydrophobicity, initial concentration, and wine protein composition were key factors. Low bentonite doses (20 g/hL) preserved the most aroma compounds.
Sanborn et al. [103]	Cv. Chardonnay and cv. Gewürztraminer wines	Sodium bentonite 100 g/hL	Bentonite in Chardonnay mainly reduced ethyl dodecanoate, leaving most VOCs unchanged, whereas Gewürztraminer exhibited notable decreases in benzeneethanol, 2-phenylethyl acetate, linalool, nerol, and long-chain ethyl esters. No sensory differences detected by trained or untrained panels.
Armada & Falqué [101]	Cv. Albariño grape must	Bentonite (type not specified) 60 g/hL	Bentonite fining decreased monoterpenes and C-13 norisoprenoids by approximately 13%, markedly diminishing linalool, geraniol, β-pinene, and limonene. C6-compounds were reduced to 33% compared to control. Alcohols, fatty acids, esters, and acetates remained unchanged with bentonite treatment.
Moio et al. [100]	Cv. Falanghina grape must	Bentonite (type not specified) 80 g/hL in combination with potassium caseinate (60 g/hL), gelatin (30 g/hL), silica gel (10 g/hL), and charcoal (20 g/hL)	Combined bentonite, casein, silica gel, and activated charcoal treatment decreased glycosidic-bound aroma in Falanghina must by up to 33% versus spontaneous or enzyme-assisted settling, particularly affecting linalool, geraniol, benzyl alcohol, 2-phenylethanol, and eugenol. This lowered free VOCs in wines, despite minimal changes in free terpenols in the grape must.

## 6. How Bentonite Influences Phenolic Compounds

Wine phenolics pertain to two major subclasses: flavonoids (e.g., anthocyanins, flavanols, flavonols) and non-flavonoids (e.g., phenolic acids and stilbenes) [111]. Flavonols and hydroxycinnamic acids contribute to white wine color [112], while flavanol monomers, dimers, and oligomers primarily influence bitterness, and flavanol polymers significantly impact astringency [113]. Phenolic compounds are crucial in white wine mouthfeel [114], with their interactions with alcohol and acidity shaping sensory attributes [115,116]. In addition to their sensory role, phenolics serve as antioxidants, enhancing wine stability and longevity [117] and acting as free radical scavengers in the human body [118].

Table 2 synthesizes the principal findings available in the literature concerning the influence of bentonite on phenolic compounds.

The use of bentonite can affect the phenolic profile and content of wine and, consequently, all of the related quality attributes discussed above. However, the underlying mechanisms remain poorly understood, particularly whether phenolic removal results from direct adsorption onto bentonite or from indirect interactions mediated by protein-phenolic complexes. Theoretically, bentonite’s ability to remove organic molecules is limited [119] due to its hydrophilic clay surface [120]. Consequently, phenolic removal is more plausibly mediated by proteins. Protein deposition on bentonite alters its surface properties, facilitating the adsorption of hydrophobic molecules and enabling multilayer adsorption [121]. Dordoni et al. [47] identified a correlation between protein and phenolic removal, reinforcing the role of proteins in this process. The binding between proteins and phenolic compounds is driven by hydrophobic interactions [66]. Specifically, the correlation between protein removal and phenolic decrease was stronger at lower pH levels (R^2^ = 0.76 and 0.79 at pH 3.00 and 3.17, respectively) than higher pH levels (R^2^ = 0.47 and 0.13 at pH 3.30 and 3.60, respectively). This supports the suggestion, mentioned in Section 3.3, for which lower pH promotes protein unfolding [17], increasing surface area and hydrophobic site exposure, thereby enhancing interactions with phenolics. Pargoletti et al. [34] demonstrated, in a model solution, that phenolic compounds bound to egg albumin through hydrogen bonds, with their removal by bentonite increasing from 19% in the absence of egg albumin to 60% in its presence. Despite uncertainties regarding the specific mechanisms, bentonite’s impact on phenolics is well documented. Lukić et al. [6] reported that all bentonite-treated wines exhibited significantly lower total phenol concentrations compared to the control, with effects varying among phenolic classes and bentonite types, in agreement with Arenas et al. [69] and He et al. [26]. Dordoni et al. [47] reported similar variability, and, based on the findings of Dordoni et al. [24], they concluded that phenolic removal is primarily associated with clay characteristics. A more negative surface charge density correlated with greater phenolic adsorption, even in the absence of proteins, thereby confirming the possibility of direct removal; conversely, bentonites exhibiting a lower surface charge showed reduced removal of phenolic compounds. In the study by Lukić et al. [6], bentonite-treated wines also contained higher hydroxycinnamoyltartaric acids (HCTAs) levels, whereas untreated controls exhibited more free forms. This aligns with findings by Horvat et al. [90] and Lukić et al. [112], suggesting that bentonite inhibits or removes the hydrolytic enzymes responsible for cleaving phenolic moiety from tartaric acid [86,104,122]. Given that hydroxycinnamic acids are precursors to the volatile phenols associated with off-flavors, this represents a potential advantage of bentonite use. Lukić et al. [112] reported the greater preservation of hydroxybenzoic acids and taxifolin in untreated controls, while bentonite-treated wines showed the highest levels of trans-piceid, consistent with Horvat et al. [90]. Additionally, bentonite addition reduced total flavonoids by approximately 30% during fermentation, increasing to nearly 50% post-fermentation, underscoring the significance of timing in bentonite treatment. This contradicts Horvat et al. [90], who reported no effect of the timing addition on phenolics. Interestingly, total phenolics remained largely unchanged, likely due to the Folin–Ciocalteu reagent’s low specificity. This observation also implies that bentonite shows a stronger affinity for flavonoids compared to other phenolic compounds, although monomeric and dimeric flavanols are only slightly or not affected [6,69,90,112]. Lagarde et al. [123] confirmed the removal of flavanols, monomers, dimers, and tannins by bentonite. However, when comparing total tannin concentrations in wine before and after treatment, no significant differences were observed, likely due to the insufficient impact of the treatment. Nonetheless, when assessing the resolubilized fraction from the precipitate, significant differences emerged, suggesting the importance of the analytical approach in drawing accurate conclusions.

**Table 2:** Extraction table of the main findings on the effects of bentonite on total (TPC) and individual phenolic compounds.

Author (Year)	Matrix	Bentonite Type and Dosage	Main Findings
Lukíć et al. [6]	Cv. Malvazija istarska grape must near the end of the fermentation and wine	Granular activated sodium bentonite (GSAB) 95 g/hL, activated sodium bentonite (PEN) 95 g/hL, activated sodium bentonite with specifically adsorbed silica and activated silica (MVC) 143 g/hL, active sodium-calcium bentonite (PUR) 238 g/hL	Bentonite partial clarification reduced TPC, especially in PUR. MVC, PUR, and PEN lowered p-hydroxybenzoic acid; PEN also reduced 2,5-dihydroxybenzoic acid. All bentonites decreased ferulic acid, p-coumaric acid, catechin, and tyrosol, but increased trans-coutaric acid, particularly in PUR. The control exhibited lower concentrations of hydroxycinnamate tartrate and higher ones of free hydroxycinnamic acids. Flavan-3-ol levels varied: the control had the highest catechin content, and PUR showed a slight reduction in procyanidin B2. Additional clarification reduced phenolics in the control, whereas fully stabilized treatments remained unchanged.
Lukíć et al. [112]	Cv. Malvazija istarska grape must near the end of the fermentation and wine	Granular activated sodium bentonite 95 g/hL	Bentonite treatment lowered protocatechuic, *p*-hydroxybenzoic, 2,5-dihydroxybenzoic acids, but increased coutaric, caftaric, and fertaric acids, with free forms more abundant in the control. Taxifolin remained higher in the control, whereas *trans*-piceide increased in the treated samples. Total flavonoids (TF) decreased by approximately 30% with bentonite, although TPC was not significantly altered. After further stabilization, TF dropped by approximately 50% in both the control and treated wines, whereas TPC content rose.
Arenas et al. [69]	Cv. Albariño wine	Sodium bentonite 120 g/hL, calcium bentonite 120 g/hL	Bentonite had minimal effects on total phenols, flavonoids, and non-flavonoids in the wine without pre-fermentative maceration (except sodium bentonite), but caused significant changes in the wine with pre-fermentative maceration, regardless of bentonite type. Individual phenolic compounds remained largely unchanged, but TPC was lowered in bentonite-treated samples in both wines.
Pargoletti et al. [34]	Model wine solution	Four different activated bentonites 40 g/hL	Bentonite preferentially adsorbs catechin and epicatechin, removing 60% of phenolics in a wine-like system with albumin (vs. 19% without albumin).
He et al. [26]	Cv. Chardonnay and cv. Sauvignon blanc wines	Calcium bentonite (PCT), sodium-calcium bentonite (BTL), sodium bentonite (PBN), calcium-sodium bentonite (SPM). 50 g/hL on Chardonnay, 30 g/hL on Sauvignon blanc	Bentonite reduced TPC in all wines. In Chardonnay, all treatments decreased caffeic acid, *p*-coumaric acid, and gallocatechin. In skin-macerated Sauvignon blanc wine, gallic, vanillic, caffeic, caftaric acids, and flavonols were lowered, especially with BTL; SPM produced the lowest caftaric acid and flavanol contents.
Horvat et al. [90]	Cv. Malvazija istarska grape must before/during fermentation and wine	Granular activated sodium bentonite 100 g/hL	Bentonite altered individual phenols, generally reducing hydroxybenzoic acids. Gallic acid was highest in the control, but decreased with additional fining, particularly in mid- or late-fermentation treatments. Protocatechuic acid increased after additional bentonite. Wines fermented with bentonite retained more caftaric, coutaric, and fertaric acids, but fewer free forms. Additional bentonite lowered coutaric and caftaric. Flavanols differed minimally across treatments, tyrosol remained unchanged, and taxifolin decreased in bentonite-treated wines. Dosing time had no effect.
Dordoni et al. [47]	Cv. Erbaluce wine with modified pH values (3.00, 3.17, 3.30, 3.60)	Four different activated sodium bentonite (GW, TG, PN, and PW) 100 g/hL	Polyphenol removal varied with pH and bentonite type. At pH 3.00 and 3.17, GW and PN treatments yielded the lowest polyphenol contents, with removal correlating strongly with protein reduction. At pH 3.30 and 3.60, TG and PW treatments retained more polyphenols. PN still removed polyphenols effectively, despite minimal protein loss.

## 7. Bentonite and the Elemental Fingerprint of Wine

Vine roots absorb soil elements, transferring them to grapes, which release them into wine during vinification [49]. The elemental content in wine primarily derives from soil, influenced by their distribution and bioavailability, grape variety, ripeness, and climate, with secondary sources including viticultural practices, enological processes, and pollution [49,50,124,125,126].

Wine contains major elements like calcium, potassium, magnesium, and sodium at 10–1000 mg/L, minor elements like aluminum, iron, copper, rubidium, strontium, and zinc at 0.1–10 mg/L, and trace elements such as barium, cadmium, cobalt, chromium, lithium, nickel, lead, and vanadium at 0.1–1000 μg/L [49]. Elemental composition is paramount for varietal and geographical origin identification, authenticity verification, and detecting counterfeit wines [126,127,128,129], thanks to the specific relationships within the soil-grape–wine system [130].

Besides identification, wine’s elemental composition influences its qualitative and sensory characteristics, like aroma, freshness, flavor, color, taste, turbidity, redox potential, and pose health risks [49,50,124,130,131,132]. For these reasons, there is a growing interest in monitoring elemental content [36,49], driven by advancements in trace element detection and rising reports of metals in the food chain [133]. The International Code of Oenological Practices [134] sets the maximum acceptable limits of various substances, including some elemental (arsenic, boron, bromine, cadmium, copper, and zinc) content in wine, to protect organoleptic qualities and ensure consumer safety [50].

Bentonite treatment influences the elemental composition of wine [29]. Its effects vary depending on the bentonite type and the wine’s characteristics, with significant variability even within the same bentonite type [135]. Element release does not directly correlate with the raw material composition of bentonites [36]. Temerdashev et al. [136] classified bentonites into distinct groups based on their composition: sodium montmorillonite, sodium–calcium montmorillonite, and montmorillonite forms containing quartz and calcite, observing that wines could be differently affected by bentonites within the same group, highlighting the complexity of these interactions. Studies indicate that higher CEC and SSA are associated with greater element exchange [27,36]. Although pH influences element release, the impact of lower or higher pH conditions remains unclear [24,27,28]. Importantly, to ensure consumer safety, bentonites must comply with legal limits on the content of some elements reported in the Monograph in the International Oenological Codex (COEI-1-BENTON: 2011) [33], as established for the European Union by the Commission Delegated Regulation (EU) 2019/934.

A detailed analysis reveals that element exchange between clay and wine varies significantly depending on bentonite type and wine composition, with calcium, sodium, magnesium, iron, aluminum, barium, and cadmium being the most released elements [24,27,34,131,136]. Conversely, potassium, copper, zinc, and rubidium are typically removed during the treatment [13,24,27,28]. Elemental variation ranges from 5% to 73%, depending on the bentonite used and the individual elements [136].

Bentonite also aids in removing toxic compounds [137], including pesticide residues, toxins, and heavy metals such as arsenic, lead, and cadmium [27,28,138]. Therefore, while bentonite significantly alters wine elemental composition [136], it does not compromise varietal identification or geographical origin attribution [131], as the natural variability in wine composition generally exceeds its influence [126]. Nonetheless, this variability should be considered in analytical studies to prevent misinterpretation [27,130]. Furthermore, understanding bentonite’s impact on elemental composition is crucial for accurately interpreting results, particularly when using multivariate statistical methods [27,130]. For instance, Rare Earth Elements (REEs) have been proposed as markers for geographical origin due to their stability across vintages [130,139]. However, since bentonite is a primary source of these elements in wine, their reliability for origin discrimination is undermined [135].

Bentonite is not the sole factor influencing wine’s elemental composition. Other winemaking processes also contribute to these changes (126, 130, 131]. For instance, maceration has been identified as the most significant factor affecting the elemental profile [131].

## 8. Strategies Beyond Bentonite: Adsorbent and Non-Adsorbent Approaches to Prevent Protein Haze in White Wine

Bentonite is the most widely used agent in the wine industry to address protein instability in white wines [13]. However, its undesirable effects have driven researchers to explore alternative solutions that maintain its efficacy in removing unstable proteins while minimizing its drawbacks [1,2]. Table 3 lists the proposed adsorbent alternatives to bentonite and summarizes the key findings from the most recent studies for each product. Certain alternatives, such as TiO_2_-based composite sorbents, functionalized mesoporous silica (FMS), dicarboxymethyl cellulose (DCMC), and magnetic nanoparticles (MNPs), have demonstrated promising potential in achieving complete wine stability. Others, including grape seed powder (GSP) and carrageenan, may currently primarily serve to reduce the required bentonite dosage. However, some alternatives, such as zeolite and zirconium oxide, require relatively high dosages to reach effectiveness levels compatible with industry standards. Others, like chitosan and chitin, may also entail significant costs. Additionally, certain undesirable effects have also been reported for some of these alternative agents, highlighting the ongoing challenge of developing selective solutions capable of removing only unstable proteins without affecting other wine components. While extensive data is available on all aspects of bentonite treatment, several key factors for these alternative products need further clarification before they can be recognized as valid substitutes. These include the potential release of material into the wine, overall costs, waste production, material durability, and a more precise assessment of their sensory impact on wine quality to better define benefits and limitations. Lastly, only some of the proposed adsorbents alternatives to bentonite are currently authorized under European Union legislation, while many remain at the research stage and are not yet implemented on an industrial scale [42]. Alternative strategies for preventing protein haze in wine exist that do not rely on adsorbent materials as substitutes for bentonite, but instead employ different mechanisms of action. Heat treatment [140] uses high temperatures to denature proteins [39], facilitating their removal by sedimentation or filtration. Similarly, high-power ultrasound (HPU) aims to achieve protein denaturation [141], while proteolytic enzymes (e.g., papain, bromelain, plant cysteine proteases, proctase) hydrolyze unstable proteins [142,143,144]. Another approach is ultrafiltration [145], which physically removes proteins based on molecular exclusion within the 1–500 kDa range. Additionally, protective colloids such as mannoproteins can be added to prevent protein aggregation rather than removing them [13]. Among these methods, ultrafiltration is highly effective [146], but also removes non-target compounds (e.g., aromas, polyphenols, polysaccharides) [147], altering sensory properties [148]. Currently, heat treatment, HPU, or enzymatic treatments alone do not achieve sufficient efficiency to be viable alternatives, except for reducing bentonite dosage, with heat treatment posing aroma loss risks [149]. However, combining these strategies can yield results comparable to bentonite, as seen in heat treatment with proteases (aspergillopepsins) on grape must [143] or heat treatment followed by ultrafiltration of protein-rich retentate [150], minimizing sensory differences. The effectiveness of mannoproteins varies considerably [151]: their use has been shown to improve protein stability [152], although achieving complete stabilization remains challenging.

**Table 3:** Extraction table reporting the principal alternatives to bentonite proposed so far.

Author (Year)	Alternative Product/Method	Main Findings
Ricci et al. [153]	TiO_2_-based composite sorbent material	TiO_2_ treatment, both in continuous and batch modes, significantly reduces turbidity (ΔNTU < 2) and protein content, with selective removal of PRPs, starting from turbidity levels of 3.20 ± 0.02 NTU (Müller Thurgau) and 6.87 ± 0.07 NTU (Gewürztraminer). TiO_2_ sorbent is the key factor influencing protein stability, with an optimal treatment duration of 60 min.
Marangon et al. [154]	Functionalized Mesoporous Silica (FMS)	FMS treatment can be performed either in batch or continuously by passing the wine through a FMS layer. FMS can be regenerated for reuse. The required dosage ranges from 10 to 150 g/hL. As FMS does not require preparatory steps, it can be directly added to the wine. Following treatment with FMS, the wine should be filtered at ≤0.45 µm. FMS achieved protein stabilization at dosages comparable to a sodium–bentonite, effectively removing thaumatin-like proteins and chitinases without altering key wine components or sensory properties.
Saracino et al. [155]	Dicarboxymethyl cellulose (DCMC)	DCMC effectively reduced protein content. Encruzado and Viosinho wines achieved stability at all tested doses, while Moscatel de Setúbal required >1.5 g/L of bentonite to achieve stabilization. DCMC had a lower impact on pH and phenolic content than bentonite and significantly reduced calcium levels. Both treatments influenced VOCs similarly, with PCA distinguishing treated samples from controls based on ethyl hexanoate and ethyl octanoate. DCMC performed better than bentonite at 0.5 g/L, but was less effective at higher doses. Its potential as a sustainable alternative lies in its ability to remove proteins while preserving wine composition.
Romanini et al. [156]	Grape seed powder (GSP)	Grape seed powder (GSP) reduced PRPs content by up to 57% in Semillon and 37% in Sauvignon blanc wines, decreasing heat-induced haze by 75% and 80%, respectively, at a dosage of 15 g/L. In comparison, complete protein stabilization was achieved with bentonite at much lower dosages (1.1–1.2 g/L). Unlike bentonite, GSP had minimal impact on wine composition, but increased flavonoid index and tannin concentration. Sensory analysis revealed greater color intensity, bitterness, and tropical fruit aromas in GSP-treated wines. Its effects on color, viscosity, astringency, and bitterness could be undesirable.
Mierczynska-Vasilev et al. [157]	Zeolite	Natural zeolites effectively stabilize wine proteins through cation exchange, requiring 4–6 g/L, whereas commercial bentonites required lower dosages (1–1.8 g/L), depending on the variety. They reduce potassium by over 30%, improving tartrate stability, and cause less wine loss than bentonite. Zeolites with 20–50 μm particles are most efficient, with pre-hydration enhancing performance. They do not negatively impact phenolic composition, affect organic acids similarly to bentonite, and produce more compact sediments with lower deposit volume (1% compared to 3.3–20% for commercial bentonites). Additionally, they offer potential reuse as a soil amendment.
Mierczynska-Vasilev et al. [158]	Magnetic nanoparticles (MNPs)	MNPs have demonstrated effectiveness in selectively removing haze-forming proteins from wine, varying efficiency depending on surface functionality, following a trend of COOH > POx > NH_2_. Increasing MNP concentration significantly reduced protein content, with 0.83 vol% achieving a 90% reduction in Sauvignon blanc and 1.66 vol% for Semillon, and effective clarification at 1.66 vol% and 3.13 vol%, respectively, confirmed by thermal stability tests. Zeta potential analysis revealed charge variations with pH, while metal content showed no significant increase after the treatment. The interaction mechanisms involve electrostatic and covalent bonding, depending on the surface chemistry. These results highlight MNPs as a promising alternative to bentonite for wine stabilization.
Ratnayake et al. [159]	Carrageenan	Kappa and Kappa/Iota carrageenans thermally stabilized white wines without sensory drawbacks, offering a renewable bentonite alternative. Their addition at various winemaking stages ensured heat stability, with minimal turbidity impact and improved filterability with pectinase. Preliminary trials (0.2–1.4 g/L) showed high variability, highlighting the polysaccharide structure’s role in protein adsorption. Large-scale trials required dosages in a range of 1–1.4 g/L; generally, lower doses were necessary in must or during fermentation than in wine, while bentonite required 1.5 g/L. Wines remained stable after 13 months. Carrageenans removed approximately 90% of proteins, although kN-carrageenan raised Na^+^ beyond Swiss export limits. Sensory analysis showed enhanced fruit aromas and less bitterness. Efficiency depended on type, viscosity, and solubility, with lower-viscosity variants dispersing better. Fermentation time increased slightly, and kN-carrageenan produced less lees than bentonite. The addition to grape musts could lead to filterability issues.
Colangelo et al. [19]	Chitosan	Chitosan (1 g/L) effectively reduced chitinases and enhanced the thermal stability of wine between 55 and 62 °C. It decreased tartaric and malic acids, potassium, and iron, while having a minimal impact on polyphenols and fermentative aromas. Chitosan also reduced free terpenes (except α-terpineol) and interacted with organic acids, particularly malic and acetic acids. The treatment lowered total protein content by 14% and improved tartaric stability, also reducing wine browning. Chitosan was relatively insoluble in alcohol strengths typical in white wines and showed potential as an alternative to bentonite for clarification.
Marangon et al. [61]	Zirconium dioxide	Zirconia effectively removes unstable proteins, reducing turbidity without significantly altering physicochemical parameters, except for total acidity at higher dosages. An alternative application using encapsulated zirconia pellets simplifies recovery without filtration or centrifugation. The material remains effective for at least 11 reuse cycles. Protein removal depends on pellet quantity and wine type. Sensory evaluation showed reduced acidity perception and occasional sulfite-like aroma due to Cu and Fe removal. Zirconia is a promising alternative to bentonite, achieving full stabilization in two out of three wines, with high-protein wines (>31 mg/L) requiring a dose of 25 g/L.
Silva-Barbieri et al. [83]	Zirconia-alumina composite (ZrO_2_/Al_2_O_3_)	At 50 g/L, zirconia–alumina composites prepared by wet impregnation and thermal calcination at 750 °C removed up to 44% of unstable proteins (TLPs, LTPs, β-1,3-glucanases), reducing turbidity by more than 50% (from 42 to 18 ΔNTU), but without achieving full stability. No major effects were observed on ethanol, glycerol, or acetic acid, although total polyphenols decreased by 14–18% and glucose was reduced. At present, these materials may represent a viable alternative (cost-effective and sustainable) only in combination with other technologies, as complete protein stability cannot be achieved with them alone.
Vincenzi et al. [55]	Chitin	Chitin has demonstrated potential as an alternative to bentonite for protein stabilization in white wine. Increasing chitin doses reduced heat-induced turbidity by up to 80%, with a protein content reduction of less than 29%, whereas bentonite removed nearly all proteins. Chitin selectively adsorbed instability-related proteins, particularly class IV chitinases. Chitin treatment also reduced polyphenol content by 25% at the highest dose (20 g/L). The efficiency ratio (turbidity reduction vs. protein removal) was higher for chitin (max 3.0) than for bentonite (max 1.6). Preliminary trials with immobilized chitin indicated its potential for continuous use. Overall, chitin could improve protein stability while better preserving wine sensory quality.

## 9. Conclusions

Bentonite remains the benchmark for protein stabilization in white wines thanks to its reliability, cost-effectiveness, and availability. Its efficacy in removing unstable proteins is undisputed; however, drawbacks, including lack of specificity, sensory modifications, wine losses, and environmental impact, underscore the need for more sustainable strategies.

Despite decades of research, key gaps persist: the molecular mechanisms underlying bentonite–protein-matrix interactions are not yet fully clarified; predictive tests tend to overestimate dosage; and the varietal and matrix effects remain poorly systematized. Promising alternatives exist, but cannot yet replace bentonite.

Future research should prioritize (i) standardizing predictive tests for dose accuracy; (ii) developing selective agents targeting haze-forming proteins; (iii) integrating innovative methods; and (iv) applying modeling approaches, including machine learning, to predict instability from compositional data. These efforts require a multidisciplinary approach addressing the technical, environmental, and economic dimensions.

Practically, wineries are advised to (i) apply bentonite during fermentation to reduce dosage and quality losses; (ii) test different bentonites to match wine-specific needs; and (iii) combine innovative solutions to mitigate negative impacts.

In conclusion, bentonite remains the standard for protein stabilization, but its role is expected to evolve toward a complementary tool within a diversified, sustainable strategy that preserves sensory quality while meeting sustainability goals.

## Data Availability

No new data were created or analyzed in this study.
